# Literature and Science

**Published:** 1891-06-20

**Authors:** 


### The Doctor's Armchair.

### LITERATURE AND SCIENCE.

"Science is not literature," says a newspaper critic. No! But until the stuff of which science is made, becomes a part of literature, the world in which the average man lives, will continue to be much more limited than it ought to be; and the average man's conquest of it will be so poor as hardly to be worth calling a victory at all. If the literary man denies that science is literature, it is not improper that the scientific man who loves literature should ask him for the grounds of his exclusion. What exactly is literature? The plain man, if he be hungry for knowledge, has a right to ask this question; and since literature is one of the oldest of the arts, it should be prepared to give him something like a plain and easily comprehended answer.

Literature deals with man; with men and with women; and with the behaviour of all sorts of men and women towards one another. It deals with human mind-stuff; with every form which that mind-stuff assumes, and with every result which that mind-stuff produces. Has science anything to do with the human mind? What is science but the latest product of human mental activity? Science does not consist of things, but of the intelligent knowledge of things. The globe of the earth, which Dr. Watts called a "terrestrial ball," is not science; but our knowledge that the earth is a globe, and may with strict propriety be called a "terrestrial ball"—that is science. Iron, and lead, and arsenic, and opium, and tobacco, and brandy—these things are not science; they existed when science was not; some of them when man was not. But inquiring and reasoning men have handled all these things; have searched out, so to speak, their origins, their family histories, their individual characters, their behaviour towards one another and towards other things and persons; and it is this orderly, intelligent, and thorough knowledge of all these things which constitutes the science of them.

What then makes science possible? It is the knowing mind. And what is science but the knowledge of the knowing mind? Science in ultimate analysis is neither more nor less than a form of mental activity. But literature also is neither more nor less than a form of mental activity. Where then is the difference between literature and science? And why does the one exclude the other, if it does exclude the other? There is no essential difference between science and literature, because each of them is a form of mental activity and nothing else. The only circumstance that reveals an apparent difference is the fact of their differing subject matter. Literature deals with men and mind; science deals with things, including men and mind. But inasmuch as the essence of literature is not its subject matter, but the mental activity that deals with the subject matter; and inasmuch as the essence of science is not its subject matter but the mental activity that deals with the subject matter, it is as plain as possible that in ultimate analysis both literature and science are forms of mental activity, or operations of the unit which we call mind, and that, therefore, between the two there is no essential and philosophical difference whatsoever.

Now, of what possible consequence is it to the practical man whether literature and science are one and the same or whether they are exactly at the antipodes of each other? It is of infinite consequence. The practical man is always a reasoner, and the reasoner is always a practical man. Every legitimate process of reasoning, every just idea, every true flight of the poet, every wholesome inspiration of the man of genius is endowed and weighted with practicality for some region of moral or material action. The literary man has hitherto led the world, and he may continue to lead it. The scientific man has never led the world until his science has become a part of literature. The scientific man never will lead the world in any greater measure than in the degree in which his science becomes a part of literature. Literature is to science what the great general is to the army; it turns the parade ground into a battle field, and the battle into a victory and a conquest. The things which constitute the subject matter of science are like the potential raw recruits who are still at the plough and in the workshop: the scientific man who has no literary faculty is the industrious sergeant who gathers the recruits together and teaches them the goose-step and company drill. But if ever the raw recruits and the drilled troopers of scientific facts and principles become victorious it is because some man of literary faculty has taken them in hand and led the imperial eagles to their world-conquering work.

Will any one affirm of Huxley that he is not a man of science? And will any one deny that he is a man of literature? There are hundreds of people who have as much knowledge as Huxley; but because they cannot give their knowledge the vitality of literature, it is like gold still buried in the mine, and comparatively useless to the world. But will anyone say of Huxley that his science is not literature? He who would so speak would prove that he had never yet shown any sort of real capacity for comprehending what literature in its essence might be. Another very familiar example occurs to us in the person of the late Mr. Darwin. If Mr. Darwin was not a literary man, what was he? And if his works are not literature, what are they? Let them be tried by any test we may choose, and they will always be found floating on the clearest surface of the literature of their period. Perhaps the most striking test that can be proposed for the literary character of any book is the influence that it has upon the thought of the time in which it is written. What was the influence of Darwin's works when they appeared upon the thought of every class of thinkers and writers in the world? It was revolutionary! Darwin made an epoch: his name formed the root of a new philosophical term. "Darwinism" has long connoted, and still connotes, a large and freshly painted target at which thousands of literary and philosophical archers are still shooting their variously feathered arrows every year.

Why do literary men of the less philosophic sort say that science is not literature? They say it because they are painfully conscious of their own ignorance of science and of the manifold disabilities which they labour under in consequence. They have reigned supreme for ages, and they fight for their threatened throne. We have shown that it is their business to reign supreme. Literature in its own nature is the Royalest of all Royalties. But no king can long continue to reign who opposes a mob of savages armed with clubs to a disciplined army scientifically accoutred and trained. The literary man, if he is to hold his throne, must make himself broadly and deeply scientific. That is the indispensable condition of enduring rule. The common world nowadays has its mental house furnished with crude scientific furniture; and its daily talk is mixed with half understood, but wholly accepted, scientific dogmas and facts. The literary man can no more hope to guide the thought of the world without assimilating the science of the times than the European missionary can hope to convert the African to a new faith without learning his language. If it be in any sense true that science is not literature, then the most imperative duty of the moment for both literary men and scientists is to convert it into literature with all possible speed.

